# Heat stress during seed development leads to impaired physiological function and plasticity in seed oil accumulation in *Camelina sativa*

**DOI:** 10.3389/fpls.2023.1284573

**Published:** 2023-11-24

**Authors:** Satya Swathi Nadakuduti, Larissa C. Laforest, Megan Tachev, Amanda N. Decker, Andrew Ogolla Egesa, Ashkon S. Shirazi, Kevin Begcy, Paul J. Sarnoski, C. Robin Buell

**Affiliations:** ^1^ Environmental Horticulture Department, University of Florida, Gainesville, FL, United States; ^2^ Plant Molecular and Cellular Biology Program, University of Florida, Gainesville, FL, United States; ^3^ The Student Science Training Program, Center for Precollegiate Education and Training, University of Florida, Gainesville, FL, United States; ^4^ Food Science and Human Nutrition Department, University of Florida, Gainesville, FL, United States; ^5^ Department of Crop and Soil Sciences, University of Georgia, Athens, GA, United States; ^6^ Center for Applied Genetic Technologies, University of Georgia, Athens, GA, United States; ^7^ Institute of Plant Breeding, Genetics and Genomics, University of Georgia, Athens, GA, United States

**Keywords:** *Camelina sativa*, heat stress, seed oil, triacylglycerol, omega-3 fatty acid, photosynthesis, seed yield

## Abstract

*Camelina sativa*, a member of the Brassicaceae, is a low-cost, renewable oilseed crop that produces seeds up to 40% oil by weight with high potential for use in food, feed, and biofuel applications. Camelina seeds contain high levels of the fatty acids α-linolenic acid (C18:3), linoleic acid (C18:2), oleic acid (C18:1), and gondoic acid (C20:1), which have high nutritional and industrial value. The impact of climate change, especially increased frequency and amplitude of heat waves, poses a serious threat to crop productivity. In this study, we evaluated the effect of elevated temperatures post-anthesis on the developing seeds of *C. sativa* and performed physiological, morphological, and chemical characterizations at 7, 14, 21, and 28 days post-anthesis (DPA), as well as at maturity. While the seed oil accumulation peaked at 21 DPA under control conditions, reaching 406mg/g dry weight, under heat stress it was only 186mg/g. Physiologically, transpiration rate (E) and internal CO_2_ concentration (Ci) increased between 2 to 9 days post-stress imposition and overall net photosynthesis was impaired. Seed yield, seed weight, and oil content reduced by 84.5%, 38.5% and 54.1% respectively. We demonstrate that post-anthesis heat stress causes severe yield losses and developmental plasticity in fatty acid accumulation in oilseeds.

## 1 Introduction

*Camelina sativa* (L.) Crantz is an oil seed crop within the Brassicaceae family and produces seeds with up to 40% oil by weight (Rodríguez-Rodríguez et al., 2013; Berti et al., 2016). Camelina is a low-cost renewable crop with high potential for use as a biofuel, in addition to multiple uses in food, feed, and other biological applications. Not only does camelina have a short life cycle (85 to 100 days), it has the potential to expand crop production areas due to its ability to grow in semi-arid and drought prone areas with relatively infertile soils and minimal agricultural inputs (Vollmann and Eynck, 2015; Bansal and Durrett, 2016; Malik et al., 2018; Cappelli et al., 2019; Von Cossel et al., 2019; Zanetti et al., 2021). Due to its high yield, high seed oil levels with favorable fatty acid compositions, low production input costs, and amenability to genetic engineering, camelina is viewed as an optimal oilseed crop for biodiesel production (Stamenković et al., 2023). Seed development in oil seed crops especially in Brassicaceae has been characterized by three phases, I) growth phase, where rapid cell division occurs, II) accumulation phase, where seed oil and protein deposits are rapidly synthesized, and III) desiccation phase, where dehydration and seed maturation occurs (Gurr, 1980; Rodríguez-Rodríguez et al., 2013).

The major components of camelina seed oil are acyl lipids stored in the form of triacylglycerols (TAGs). TAGs are esters of glycerol, with each hydroxyl group esterified with a fatty acid (FA) at all three carbons. Due to their highly reduced state, TAGs represent compact molecules for carbon and energy storage in living organisms. *De novo* FA biosynthesis in plants occurs within the plastids, where acetyl CoA is used as the carbon precursor for the assembly of FAs. Acyl groups attached to acyl carrier proteins are elongated by sequential addition of 2 C units (Li-Beisson et al., 2013). The biosynthesis of TAGs occurs at the endoplasmic reticulum by sequential acylation and subsequent dephosphorylation of glycerol-3-phosphate (Li-Beisson et al., 2013). Diacylglycerol represents an important branch point between the synthesis of TAGs and membrane lipids. The last step in TAG synthesis involves the acylation of diacylglycerol using acyl CoAs or phosphatidylcholines as acyl donors (Li-Beisson et al., 2013; Mueller et al., 2017). Phosphatidylcholine is not only a major metabolic precursor for TAG synthesis but is also an abundant membrane lipid. The major flux in TAG synthesis (>95%) is associated with the diacylglycerol/phosphatidylcholine intermediate pool (Pollard and Shachar-Hill, 2022). Camelina TAGs have a high percent of polyunsaturated fatty acids (PUFAs) such as ω-3 FA, α-linolenic acid (C18:3, 19-43%) and ω-6 FA, linoleic acid (C18:2, 11-28%) (Vollmann and Eynck, 2015; Zanetti et al., 2017; Anderson et al., 2019; Hotton et al., 2020; Zanetti et al., 2021)) together constituting up to 50% of total fatty acids in camelina seed, followed by monounsaturated FAs (MUFAs).

Climate change is typically accompanied by an increase in the frequency, duration, and amplitude of droughts and heat waves (Zandalinas et al., 2021). Temperature is a major environmental factor that acts as an abiotic stressor; impacting plant growth and posing a major threat to crop productivity. High atmospheric temperatures have profound effects on the internal thermal environment of plants, altering the major physiological processes that dominate carbon fluxes, including photosynthesis, photorespiration, and respiration. In addition, a suite of stress-responsive organic compounds are formed under high temperatures, which consume substantial amounts of carbon (Dusenge et al., 2019). High temperatures have a detrimental effect on plant development, particularly on reproductive stages (Chen et al., 2016; Begcy et al., 2019b), impacting seed production and thus crop productivity. At the physiological level, high temperatures negatively affect the activity of photosynthetic enzymes (Jensen, 2000; Parrotta et al., 2020; Moore et al., 2021). Particularly, RUBISCO activity and its affinity for CO_2_ decreases as temperature increases, affecting the overall plant photosynthetic capacity (Salvucci and Crafts-Brandner, 2004). Under heat stress (HS), an increased transpiration rate in response to high temperature works as an evaporative cooling mechanism to prevent thermal damage, a necessary trade‐off with water conservation (Deva et al., 2020). However, excessive loss of water through elevated transpiration may surpass the plant’s water uptake and transport, leading to wilting and closing of the stomata. The resulting decrease in CO_2_ uptake has a clear effect on the net photosynthetic rate, and when accompanied by degradation of proteins and pigments due to heat damage, electron transport is impaired and photosystem I (PSI) and PSII are inactivated (Iqbal et al., 2021).

Plants have developed several adaptive responses to elevated and varying temperatures, including improved water use efficiency, decreased growth, early flowering, and modulation of plant membranes and storage lipids by triggering lipid-dependent signaling cascades (Zheng et al., 2011; Hou et al., 2016; Zhang and Sonnewald, 2017). As a result, investigating plant physiological responses to changing environmental factors, particularly from floral transition to seed set, is integral in adapting crop plants to combat for climate change. Specifically, high temperatures drastically affect plant reproductive stages impacting seed set, grain fill, and yield (Folsom et al., 2014; Lohani et al., 2022; Magno Massuia de Almeida et al., 2023) in addition to seed composition and quality (Elferjani and Soolanayakanahally, 2018; Alsajri et al., 2020; Brock et al., 2020; Ortiz et al., 2022). Although camelina has broad climatic adaptability, the resulting impact on seed oil yield and fatty acid profiles is key to its utility as a sustainable crop. Though limited agronomic studies have been performed in camelina, it is clear that temperature impacts oil yield and fatty acid composition of oil seeds (Vollmann and Eynck, 2015; Obour et al., 2017; Raziei et al., 2018; Krzyżaniak et al., 2019; Brock et al., 2020). Floral development and seed yield are strongly affected by temperature, with milder temperatures resulting in higher yields (Krzyżaniak et al., 2019; Zanetti et al., 2021). Furthermore, camelina accessions and cultivars grow in varying climatic niches throughout the globe, and temperature was found to elicit plasticity in seed oil in camelina (Brock et al., 2020) and other oil seed crops (Izquierdo et al., 2016; Xu et al., 2016; Alsajri et al., 2020; Nakagawa et al., 2020). *C. sativa* includes spring and winter biotypes, with spring biotypes being the most studied thus far since winter biotypes required vernalization for flowering (Anderson et al., 2018; Malik et al., 2018). The majority of camelina accessions available and analyzed are spring biotypes (Hotton et al., 2020) and biochemical characterization of seed oil from multiple accessions indicated that the average FA profile has low levels of saturated FAs and higher levels of PUFAs (Rodríguez-Rodríguez et al., 2013; Hotton et al., 2020). Further, the predominant FAs and their proportions in total oil are consistent even among winter biotypes (Rodríguez-Rodríguez et al., 2013; Hotton et al., 2020). In the present study, we have chosen Suneson, a spring cultivar that is extensively used in biotechnology and genetic studies (Na et al., 2018; Ozseyhan et al., 2018; King et al., 2019; Na et al., 2019; Lhamo et al., 2020; Gomez-Cano et al., 2022; Bengtsson et al., 2023). We performed physiological, morphological, and chemical characterization of multiple camelina seed developmental stages to investigate the impact of post-anthesis HS on seed development and fatty acid accumulation.

## 2 Materials and methods

### 2.1 Plant material and growth conditions

*Camelina sativa* cv. Suneson plants were grown in a controlled environment growth chamber facility starting from seed at temperatures of 22/18°C (light/dark), 40% relative humidity with a photon flux density (PPFD) of 300 µmol m^-2^ s^-1^ under a 16h photoperiod. Flowers were marked at anthesis, and seeds were harvested for analysis at 7 days post anthesis (DPA), 14 DPA, 21 DPA, 28 DPA, and at full maturity directly into liquid nitrogen and stored at -80°C until processed. Mature seeds were collected when siliques dried out, and before seeds shed upon pod dehiscence. Anthesis was defined as a stage with fully opened flowers, with all four petals in bright yellow color. Anthesis was marked for a total of 55 plants in the CO growth chamber. When the plants developed at least 10-12 flowers at anthesis, half of the plants were either retained at the CO or moved to the HS growth chamber for seed development. The HS growth chamber was maintained at 34/24°C (light/dark) with all the other environmental conditions identical to the CO growth chamber. Five biological replicates for each stage were collected for all analyses. In the case of 7 DPA and 14 DPA, a combination of 3-5 plants were used to pool seeds as one replication due to small size of the seed with high water content.

### 2.2 Plant physiological measurements

The uppermost, completely expanded leaf of plants under CO and HS conditions was used to measure photosynthetic rate (A), transpiration rate (E), intracellular CO_2_ concentration (Ci) and stomatal conductance (gws) using an infrared gas analyzer (Li-COR Li 6800, Lincoln, NE) as previously described (Begcy et al., 2019a). Measurements were taken at the beginning of flowering and extended over a period of 22 days, occurring at the following time points: 0, 2, 5, 9, 12, 17, and 22 days from flower initiation.

### 2.3 Analysis of seed fatty acid composition and quantification

Seeds were removed from the siliques stored at -80°C and lyophilized in a benchtop freeze-dryer (FreeZone, Labconco, Kansas City, MO, USA) for 24h at ∼0.5mbar and -90°C. Seed lipids were quantified by converting their FAs to the corresponding fatty acid methyl esters (FAMEs) by transmethylation reaction based on (Li et al., 2006). Using a homogenizer, ~20 mg of lyophilized seeds were homogenized in 3ml toluene, combined with 1ml of 5% (v/v) concentrated sulfuric acid in methanol, 25µl 0.2% BHT (butylated hydroxytoluene in methanol), and 10µl of internal standard (IS) C17:0 TAG (5mg/ml) (Sigma# T2151). The mixture was vortexed for 30sec, then heated at 90-95°C for 1.5h and cooled to room temperature. 1.5ml of 0.9% NaCl (w/v) was added, and FAMEs were extracted 3x with 2 ml hexane. Pooled organic phases were then evaporated under N_2,_ and dried FAMEs were dissolved in 1ml of hexane. The FAMEs were analyzed by Gas Chromatography (GC 6890 Series, Agilent, Wilmington DE) using a DB-225MS column (30 m × 0.25 mm × 0.25 μm) at a flow rate of 0.8 mL/min. The GC conditions were as follows: initial temperature of 120°C, then ramp to 220°C at a rate of 4°C/min, and hold for 35 min. An external standard (Supelco^®^ 37 Component FAME Mix, CRM47885, Sigma-Aldrich, St. Louis, MO) was used for peak identification and C17:0 IS was used for quantification.

### 2.4 Determination of plant growth parameters

Twelve plants were dedicated to determining plant growth parameters, thus, excluded from any destructive harvesting of developing seeds until the final harvest. Plants were grown in the CO growth chamber until the plants had at least 10 flowers at anthesis with multiple unopened flower buds. Half of the plants were transferred to the HS growth chamber described above while the rest remained under CO conditions. Six plants per treatment were exclusively utilized to determine various growth parameters, including total seed yield, 100 seed weight, above-ground biomass, and to collect plant physiological measurements under CO and HS conditions. Plants were harvested when siliques began to crack open, and seeds reached maturity. The seeds were cleaned and weighed for the total yield per plant. 100 seeds per plant were manually counted and weighed to determine 100-seed weight. For above-ground biomass measurements, upon reaching its final stages of senescence wherein the plant material is completely dry, with all seed pods dehisced, the whole plant above the soil surface was collected into paper bags, air dried until completely dry, and dry weight was measured per plant. Seeds from CO and HS treatments were sown, with six biological replications each; 30 seeds per replication were germinated in petri plates with moist filter paper and wrapped in aluminum foil. The plates were kept under CO conditions for a week and percentage germination was calculated as percentage of number of seeds germinated (with radicle emergence after one week) ÷ total number of seeds. A similar experiment with two repetitions was conducted where 30 visibly heat damaged and regular HS seeds were germinated and percentage of germination was calculated.

### 2.5 Microscopic analysis of camelina siliques and seeds

Siliques and seeds harvested at 7, 14, 21, and 28 DPA of camelina plants grown under CO and HS conditions were imaged at 0.8-3X magnification on a Leica dissecting microscope (Wetzlar, Germany) using a microsystems CMS camera calibrated with Leica Application Suite X LAS X (3.7.4.23463).

### 2.6 Statistical analyses

Statistical analyses were performed either using JMP or R packages rstatix, ggplot, and ggsignif. Physiological and FAME composition data was subjected to a student’s *t-test* to compare differences between plants grown under CO and HS conditions. Differences were considered significant at *P* ≤ 0.05.

## 3 Results

### 3.1 Heat stress impairs physiological performance of *C. sativa* plants

**Figure 1:**
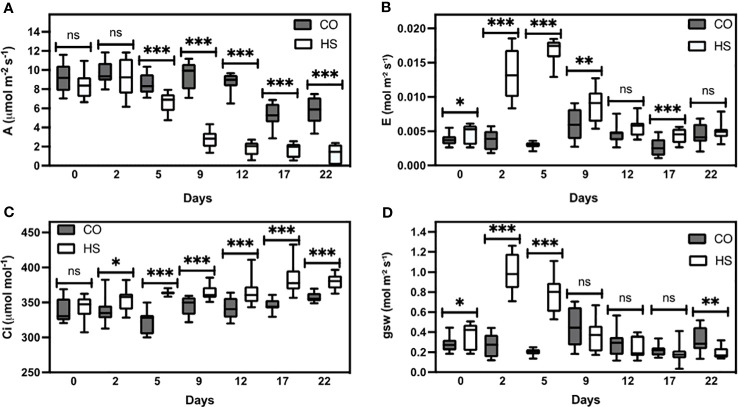
Gaseous exchange measurements of camelina under control and heat stress. **(A)** Photosynthesis (A), **(B)** transpiration rate (E), **(C)** intercellular CO_2_ (Ci), and **(D)** stomatal conductance (gsw). CO-control, HS-Heat stress conditions. Measurements were performed using 300 Photosynthetic Photon Flux Density (PPFD) at ambient CO_2_ (400 µmol mol^-1^) and 40% relative humidity. ns *P* > 0.05; *P ≤ 0.05, **P ≤ 0.01, and ***P ≤ 0.001. The error bars represent standard error of the mean.

To study the effect of increased temperature on the developing camelina siliques, we imposed a persistent HS environment for the duration of reproductive development post-anthesis. A parallel set of plants was maintained under optimal growth conditions and used as a control for all experiments. We quantified the physiological responses and observed that HS strongly impacts gas exchange parameters (**Figure 1**). Under CO (non-stressed) conditions, camelina plants did not show variations in photosynthetic rate (A), transpiration rate (E), intracellular CO_2_ concentration (Ci), and stomatal conductance (gws) (**Figure 1**). Interestingly, a minor decline in photosynthesis was observed under CO conditions on and after day 17, likely due to typical senescence initiation (**Figure 1A**). In contrast, high temperatures decreased plant overall photosynthetic rate (A; **Figure 1A**). We observed a significant increase in transpiration rate (E) in response to high temperatures, effective from day one after HS induction (*P* ≤ 0.05), peaking on the seventh day (*P* ≤ 0.001), and declining thereafter (**Figure 1B**). A similar pattern was observed for internal CO_2_ concentration (Ci) as well (**Figure 1C**). Additionally, a significant increase in stomatal conductance (gws) (*P* ≤ 0.001) was detected under HS, which mirrored transpiration (E) under HS (**Figure 1D**). While CO plants had green leaves until four weeks following the start of temperature treatments, HS plants showed accelerated senescence, 10 days earlier than CO. At the end of reproductive development, HS camelina plants showed almost a 90% reduction in photosynthetic rate compared to CO plants. Taken together, our results show that increased temperature during reproductive development negatively affects the physiological status of camelina.

### 3.2 Morphological variation during *C. sativa* post-anthesis seed development under control and heat stress conditions

**Figure 2:**
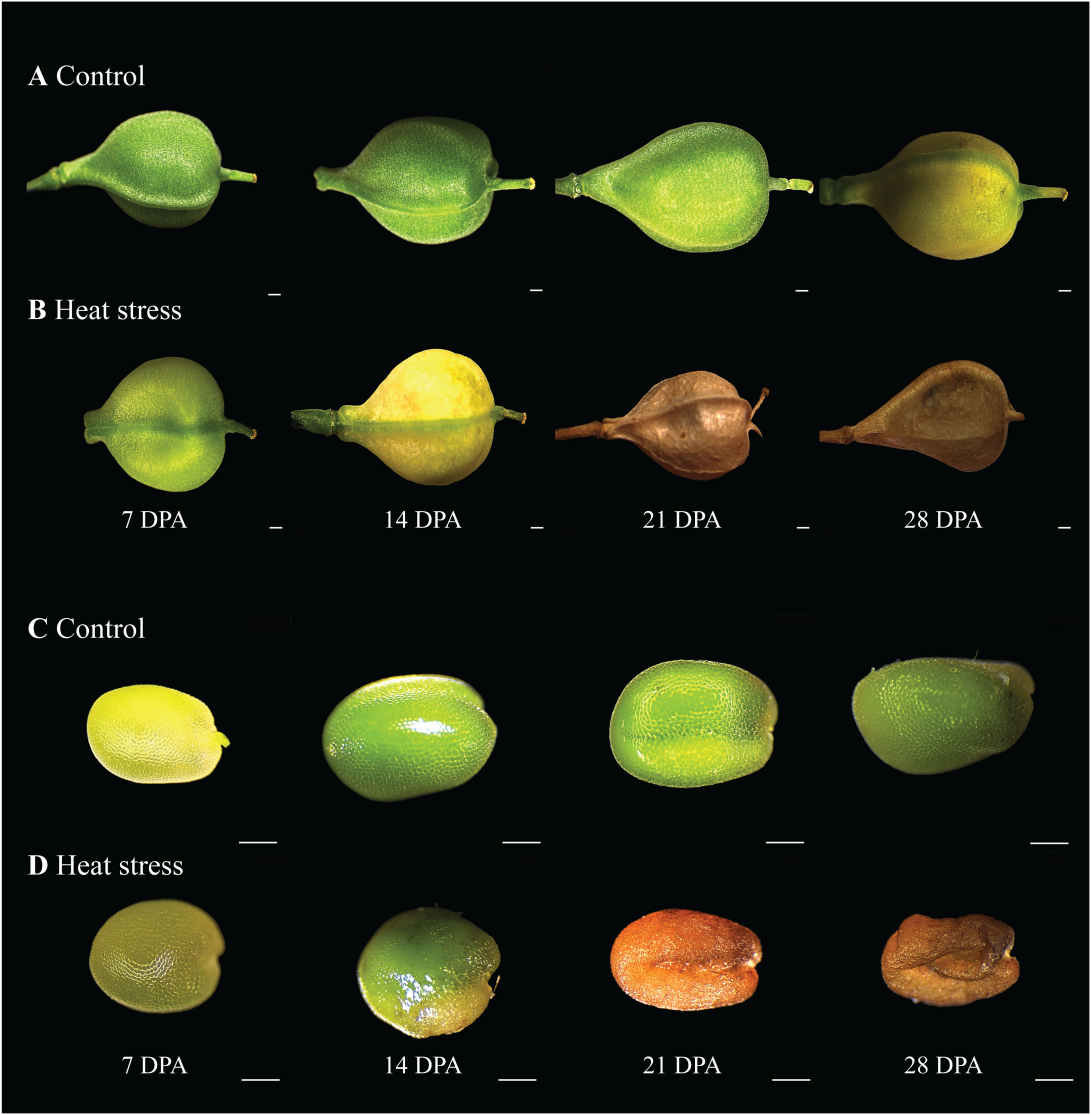
Morphological variation of post anthesis siliques and seed development. **(A)** Siliques under control conditions (CO) (22/18°C light/dark), **(B)** Siliques under heat stress (HS) (34/24°C light/dark), **(C)** CO seed, **(D)** HS seed. DPA, days post anthesis. Scale bar = 500µm.

Under CO conditions, seed development of *C. sativa* progressed from anthesis to maturity in approximately 35 days. Silique and seed development occurred mainly during the first growth phase, which lasted up to 14 DPA, after which most siliques had sizes comparable to those at maturity (**Figure 2A**). The siliques remained green in color until 28 DPA, after which slight browning began. However, under HS conditions, silique development progressed more rapidly, with browning starting at 14 DPA (**Figures 2A, B**). While seed size between 14-21 DPA under CO is comparable to mature seed (**Figure 2C**), under HS several seeds per plant had visible heat damage at all stages, most prominently from 14 DPA onwards (**Figure 2D**). In addition to impaired seed development, the filling of siliques was also affected when developed under HS resulting in hollower siliques, whilst CO siliques were full of developing seeds (**Figures 2A, B**). Seed viability was evaluated for HS and CO seeds by germination experiments. Approximately 8% of HS seeds demonstrated visible heat damage with far darker coloration and a more shriveled appearance (**Figure 2D**). Although no significant overall differences in germination percentage was observed for undamaged HS seeds and CO seeds, visibly heat-damaged seeds (**Figure 2D**) had a 90% reduction in seed germination compared to visibly normal HS seeds.

### 3.3 High temperature negatively affects content and rate of fatty acid accumulation in developing *C. sativa* seed

**Figure 3:**
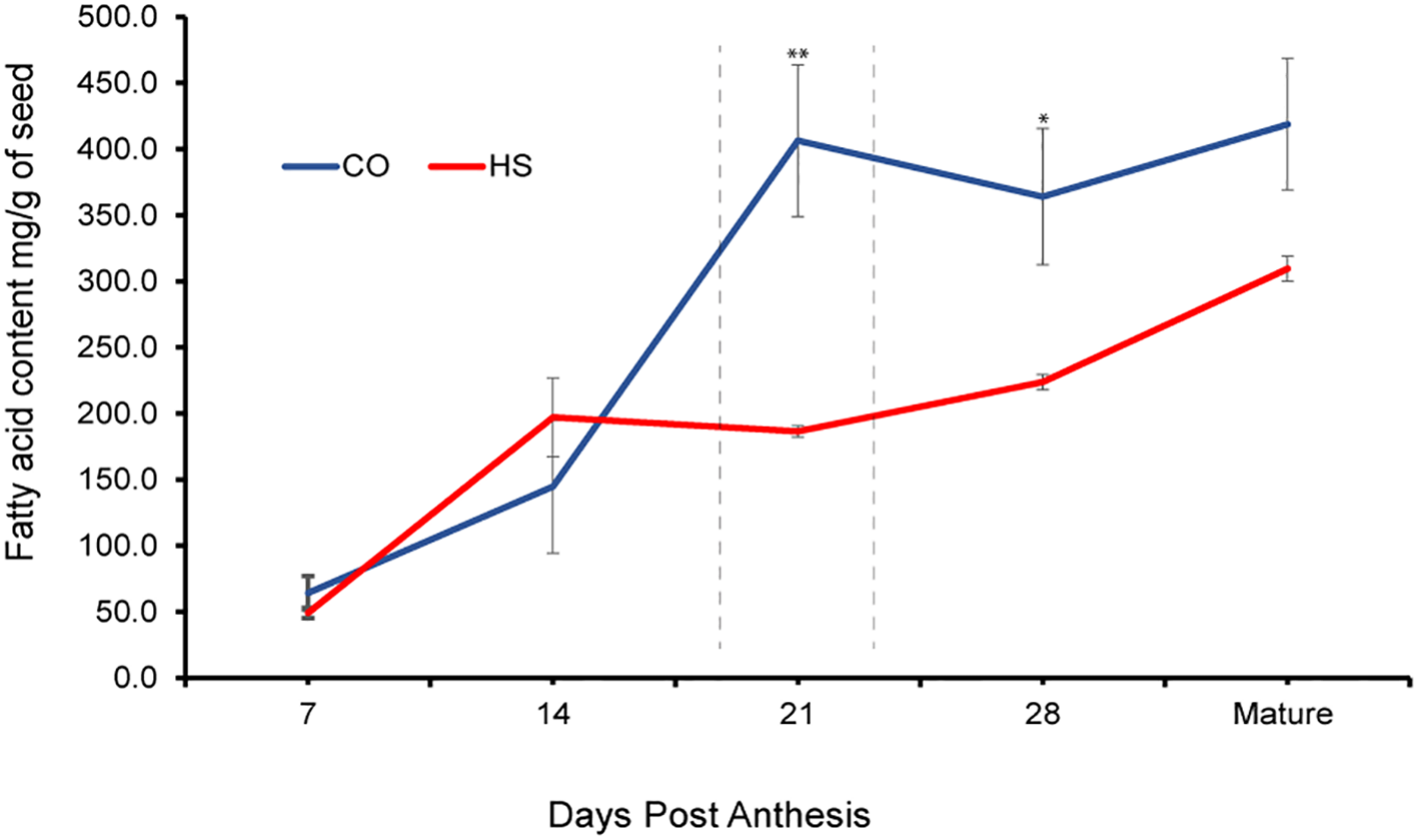
Fatty acid accumulation in developing seed of *C. sativa.* Under control (CO) and heat stress (HS) conditions at 7, 14, 21, 28 days post anthesis and at full maturity. The peak accumulation under CO conditions is designated with a dashed line. Asterisks denote statistically significant differences between the control (CO) and heat stress (HS) conditions at the same seed developmental stage (*P ≤ 0.05, **P ≤ 0.01, and ***P ≤0.001) as determined by Student’s *t* tests. The error bars represent standard error of the mean.

To identify the effects of HS on seed oil profiles, we quantified FA accumulation throughout *C. sativa* seed development from 7 DPA, 14 DPA, 21 DPA, 28 DPA, and at maturity under CO and HS conditions. The total FA content at 7 DPA under CO and HS conditions was 64 mg/g seed and 49 mg/g seed, respectively (**Table 1**) and not significantly different in this early stage of development. However, the rate of FA accumulation varied throughout seed development under both CO and HS conditions. There was a continuous increase of FAs with no significant differences in seed FA content or composition under both treatments until 14 DPA. However, under HS, 14 DPA seeds had a slightly higher content of individual FAs detected when compared to CO (**Figure 3**) with a visible heat damage on the seeds (**Figure 2D**). FA accumulation in *C. sativa* seeds peaked at 21 DPA under CO conditions with 406 mg/g seed, and there was only a slight increase of total FA after this stage until maturity (**Figure 3**; **Table 1**). The impact of HS was most prominent at 21 DPA. Most FAs had significantly lower accumulation by 21 DPA under HS compared to CO, reducing the total seed FA content by 54% (**Tables 1**, **S1**). Unlike CO, where most of the FA accumulation occurred between 14 DPA and 21 DPA, there was a continued but low rate of FA accumulation throughout the seed development under HS after 14 DPA with the total FA content in HS matured seed reaching to 309.5 mg/g compared to 418.8 mg/g in CO seed (**Figure 3**; **Table 1**).

**Table 1:** Fatty acid composition and quantification at various seed developmental stages in mg g^-1^ from *Camelina sativa* dry seed.

FA	7DPA			14DPA			21DPA			28DPA			Mature		
mg/g seed	CO	HS	SS	CO	HS	SS	CO	HS	SS	CO	HS	SS	CO	HS	SS
C14:0	0.27 ± 0.1	0.13 ± 0.02	ns	0.17 ± 0.07	0.26 ± 0.03	ns	0.26 ± 0.08	0.2 ± 0.01	ns	0.23 ± 0.04	0.26 ± 0.01	ns	0.25 ± 0.03	0.3 ± 0.02	ns
C16:0	10.41 ± 1.09	9.78 ± 0.58	ns	15.36 ± 4.77	20.28 ± 2.29	ns	27.83 ± 3.79	15.8 ± 0.33	*	24.01 ± 3.56	18.4 ± 0.33	ns	25.52 ± 3.2	25.04 ± 0.88	ns
C16:1	0.55 ± 0.09	0.19 ± 0.08	*	0.25 ± 0.07	0.55 ± 0.07	*	0.39 ± 0.06	0.57 ± 0.02	*	0.43 ± 0.06	0.7 ± 0.02	**	0.44 ± 0.05	0.77 ± 0.03	***
C18:0	3.95 ± 1.1	2.17 ± 0.25	ns	9.61 ± 3.17	7.73 ± 0.73	ns	15.49 ± 2.11	7.28 ± 0.2	**	12.28 ± 1.86	8.47 ± 0.29	ns	12.24 ± 1.6	11.74 ± 0.76	ns
C18:1^n9c^	10.52 ± 4.74	8.77 ± 1.34	ns	29.4 ± 8.93	38.66 ± 4.66	ns	48.43 ± 6.35	38.45 ± 1.32	ns	38.53 ± 5.76	46.76 ± 1.43	ns	59.93 ± 9.81	53.01 ± 1.24	ns
C18:2^n6c^	24.82 ± 4.72	20.05 ± 1.26	ns	46.31 ± 14.36	57.15 ± 8.36	ns	92.87 ± 11.45	55.56 ± 1.34	*	72.29 ± 10.81	65.28 ± 1.78	ns	79.93 ± 9.28	89.25 ± 2.39	ns
C18:3^n3^	9.33 ± 1.99	6.24 ± 0.65	ns	24.73 ± 9.31	31.27 ± 6.21	ns	118.17 ± 19.56	27.64 ± 1.12	**	123.26 ± 17.06	33.64 ± 0.9	***	138.4 ± 15.17	62.86 ± 3.29	**
C20:0	1.36 ± 0.4	0.89 ± 0.18	ns	2.98 ± 1.33	6.58 ± 0.86	ns	10.47 ± 1.44	6.39 ± 0.19	*	8.87 ± 1.28	7.19 ± 0.25	ns	7.21 ± 0.76	9.17 ± 0.46	ns
C20:1	1.59 ± 1.42	0.21 ± 0.06	***	11.24 ± 6.53	23.99 ± 4.32	ns	59.16 ± 8.61	23.66 ± 1.01	**	51.11 ± 6.98	28.96 ± 0.95	*	62.74 ± 7.91	36.87 ± 1.06	*
C20:2	0.24 ± 0.08	0.23 ± 0.06	ns	1.25 ± 0.52	2.75 ± 0.6	ns	8.41 ± 1.31	2.65 ± 0.12	**	7.8 ± 1.11	3.24 ± 0.12	**	9.01 ± 0.95	4.77 ± 0.16	**
C20:3	0.85 ± 0.37	0.1 ± 0.04	ns	0.96 ± 0.29	0.82 ± 0.19	ns	4.7 ± 0.92	0.75 ± 0.04	**	5.5 ± 0.74	1.48 ± 0.59	**	6.01 ± 0.63	1.72 ± 0.11	***
C22:0	0.39 ± 0.09	0.37 ± 0.05	ns	0.74 ± 0.25	1.71 ± 0.2	*	2.35 ± 0.35	1.53 ± 0.04	*	2.08 ± 0.28	1.68 ± 0.05	ns	1.67 ± 0.2	2.47 ± 0.08	**
C22:1	0 ± 0	0 ± 0	ns	1.07 ± 0.96	3.95 ± 1.63	ns	14.65 ± 2.19	4.07 ± 1.03	**	13.75 ± 1.94	5.73 ± 0.23	**	12.18 ± 0.92	8.61 ± 0.34	**
C24:1	0 ± 0	0 ± 0	ns	0.57 ± 0.23	1.33 ± 0.32	ns	3.12 ± 0.54	1.83 ± 0.14	*	3.86 ± 0.55	1.99 ± 0.07	**	3.32 ± 0.24	2.89 ± 0.13	ns
Total FA	64.2 ± 12.7	49.1 ± 3.9	ns	144.6 ± 50.4	197 ± 29.9	ns	406.3 ± 57.4	186.4 ± 4.4	**	364 ± 51.6	223.8 ± 5.8	*	418.8 ± 49.9	309.5 ± 9.6	ns

DPA, Days Post Anthesis. The data represents the mean of n=5 ± SE. Asterisks denote statistically significant (SS) differences between the control (CO) and heat stress (HS) conditions at the same seed developmental stage (ns *P* > 0.05; *P ≤ 0.05, **P ≤ 0.01, and ***P ≤ 0.001) as determined by Student’s *t* tests. SE = standard error of the mean.

### 3.4 Qualitative and quantitative variation in *C. sativa* seed FA composition between developmental stages under control and heat stress conditions

**Figure 4:**
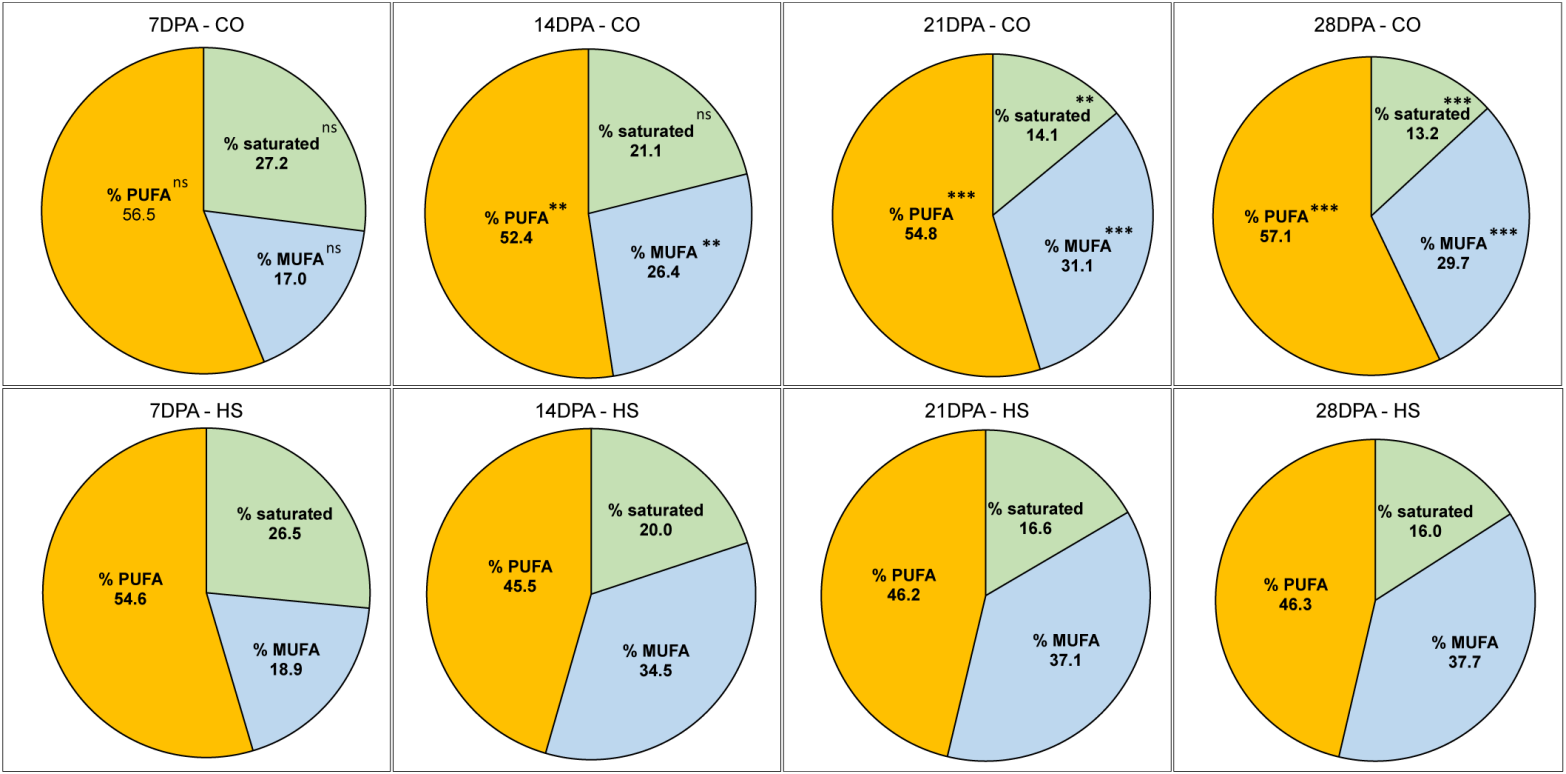
The percentage of Fatty acids (FAs) in developing seed of *C. sativa* categorized based on degree of saturation. Percentage of FAs categorized as saturated FAs, mono-unsaturated FAs (MUFAs), poly-unsaturated FAs (PUFAs) under control (CO) and heat stress (HS) conditions at 7, 14, 21, 28 days post anthesis and at full maturity. Percentage of Saturated FAs constituted sum of C14:0, C16:0, C18:0, C20:0, C22:0 FAs; % MUFAs constituted C16:1, C18:1, C20:1, C22:1, C24:1; % PUFAs constituted C18:2, C18:3, C20:2, C20:3, C20:2. Asterisks denote statistically significant (SS) differences between the control (CO) and heat stress (HS) conditions at the same seed developmental stage (ns *P* > 0.05; *P ≤ 0.05, **P ≤ 0.01, and ***P ≤ 0.001) as determined by Student’s *t* tests.

In mature *C. sativa* seeds under CO conditions, we detected 33.3% α-linolenic acid, an ω-3 FA (C18:3) and 19.2% linoleic acid (C18:2), an ω-6 FA, together constituting slightly more than half (52.5%) of total FAs (**Tables 1**, **S1**). The next two major FAs, oleic acid (C18:1) and gondoic acid (C20:1) constituted 13.6% and 14.9% respectively. Altogether, in *C. sativa* mature seed, the four major FAs, C18:3, C18:2, C18:1 and C20:1 constituted 81% of seed oil, and their contents increased from 7 DPA to maturity. The FA composition in the early stages of seed development is distinct from mature seed even under CO conditions. For example, the percentage of saturated FAs detected is the highest at 7 DPA (27.2%) and subsequently decreased as the seeds developed, with the lowest levels detected at seed maturity (13.2%) (**Table 1**; **Figure 4**). The two major saturated FAs at 7 DPA include palmitic acid (C16:0, 17.4%) and stearic acid (C18:0, 6.02%), constituting up to 82% of saturated FAs. However, as the seed matured, reduction of saturated FAs and a corresponding increase in the unsaturated FAs was observed. As the seed FA accumulation peaked at 21 DPA under CO, the composition of FAs also varied with C18:3 and C20:1 FAs increased by five times, and C18:2 FAs doubled compared to 14 DPA (**Table 1**). On the other hand, the percentage of C18:1 FA in seeds dropped from 21.9% to 12.3% within the same period.

High temperature during seed development resulted in both qualitative and quantitative changes in FA accumulation. Some notable observations are a decrease in the percentage PUFAs and an increase in percentage MUFAs under HS compared to CO in most stages of *C. sativa* seed development. For example, at 21 DPA C18:3 FA is the major FA with 118.1mg/g in CO in contrast to 27.6mg/g under HS. Although there is a continued but low rate of FA accumulation under HS, we detected that C18:3 FA was significantly lower under HS at all stages of seed development until maturity, with C18:2 being the major FA in most stages instead. The ratio of ω-6 to ω-3 is therefore elevated under HS from 21 DPA (0.7, CO vs 2.0, HS) until maturity (0.57, CO vs 1.4, HS) (**Table 1**). Other PUFAs, including C20:2 and C20:3 FAs also showed a similar trend with reduced content under HS (**Table 1**). In contrast, the percentage of C18:1 FA remained higher under HS in all stages of seed development compared to CO. For example, percentage of C18:1 FA under HS at 21 DPA, 28 DPA and maturity are 20.6%, 20.8%, and 17.1% while under CO it is 12.2%, 10.8%, and 13.6% respectively.

### 3.5 Heat stress during seed development in *C. sativa* impacts yield

**Figure 5:**
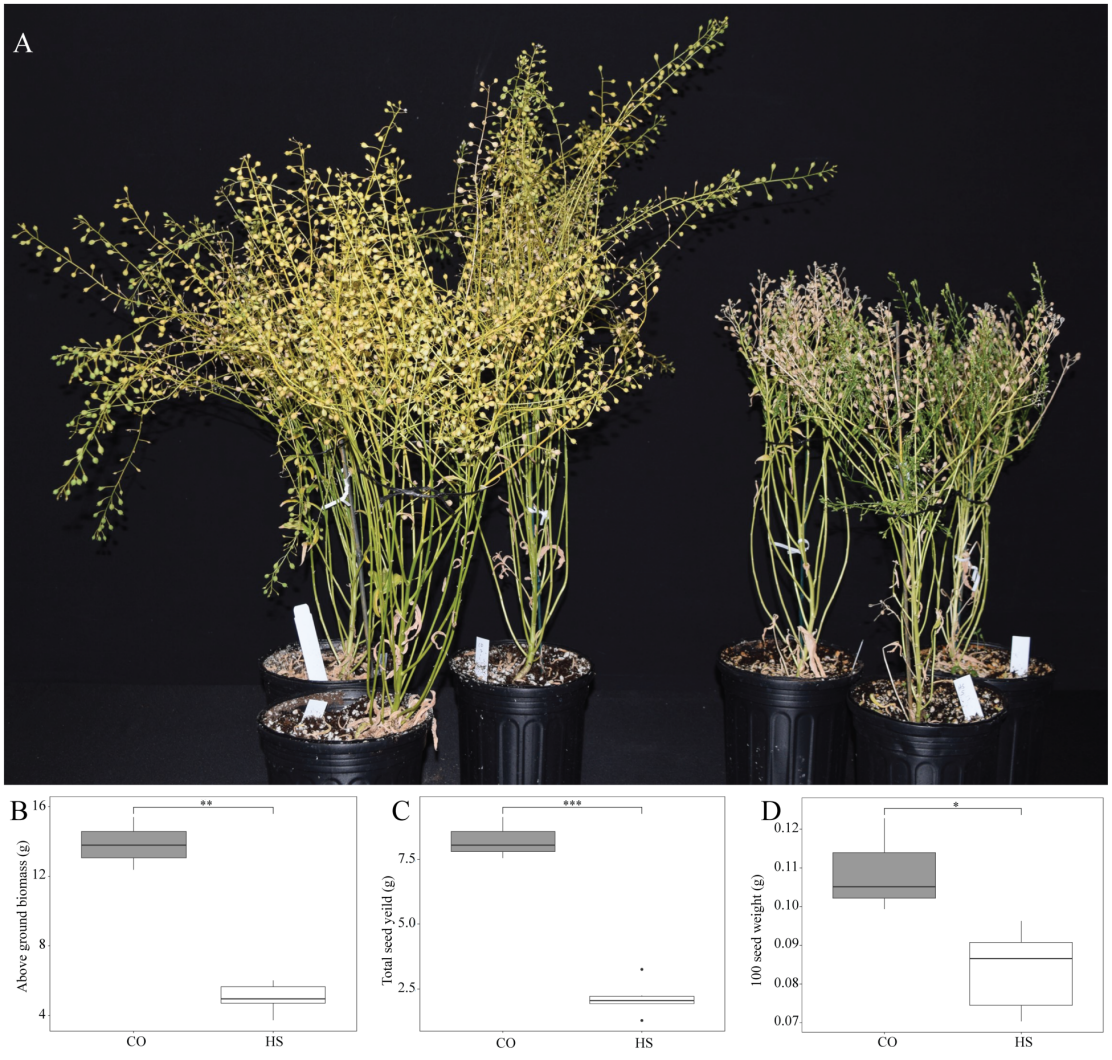
Effect of heat stress on plant growth and seed yield. **(A)** *Camelina sativa* cv. Suneson plants grown under control (CO) temperature of 22/18°C (light/dark) throughout their lifecycle compared to plants subjected to heat stress (HS) at 34/24°C during seed development phase. **(B)** 100-seed weight, **(C)** total seed yield, **(D)** Total above ground biomass. Asterisks denote statistically significant (SS) differences between the control (CO) and heat stress (HS) conditions at the same seed developmental stage (*P ≤ 0.05, **P ≤ 0.01, and ***P ≤0.001) as determined by Student’s *t* tests. The error bars represent standard deviations.

High temperatures during seed development impacted the overall growth of the camelina significantly (**Figure 5A**) with an 81.7% decrease in the above-ground biomass under HS compared to CO (**Figure 5B**). There was a substantial 84.5% reduction in seed yield in plants that continuously experienced heat during seed development (**Figure 5C**). Furthermore, the seed weight of camelina grown under HS had a 38.5% decrease in 100 seed weight (**Figure 5D**).

## 4 Discussion

### 4.1 Heat stress impairs physiological plant performance

Heat stress is one of the major environmental constraints for plant growth and agricultural crop productivity (Al-Khatib and Paulsen, 1990; Bita and Gerats, 2013). Photosynthesis is a heat-sensitive cellular process in plants since high temperatures cause damage to chloroplasts and mitochondria, inactivate RUBISCO, and reduce chlorophyll content and photosystem II (PSII) efficiency (Bernacchi et al., 2001; Bernacchi et al., 2003; Cen and Sage, 2005; Sharkey, 2005; Hu et al., 2020). One of the common strategies used by plants to tolerate HS conditions involves the adjustment between photosynthetic and transpiration rates through the regulation of stomata opening and closure. Stomata closure avoids water loss through evaporation but simultaneously reduces CO_2_ intake and thus photosynthetic rate (Greer and Weedon, 2012; Shen et al., 2017). Some heat-tolerant plants, in contrast, can maintain their physiological status for longer periods and thus keep open their stomata and CO_2_ uptake while cooling their leaf surface at elevated temperatures. This avoids, for instance, membrane and thylakoid damage. Thus, tight physiological regulation is required to balance the various effects caused by increased temperatures (Bita and Gerats, 2013; Rajendra Prasad et al., 2021). Compared with HS conditions, we observed that camelina plants grown under CO conditions have higher photosynthetic rates. Interestingly, after two days of heat treatment, higher transpiration (**Figure 1B**) and stomatal conductance (**Figure 1D**) were observed in HS camelina plants which indicates the initial reaction to open stomata to reduce the high leaf temperature and higher CO_2_ uptake (**Figure 1**). Under HS, a steady decline of photosynthesis was observed after the fifth day and reaching almost non-detectable levels after day 15 (**Figure 1A**), this is also reflected in the rapid senescence of the pods and developing seeds (**Figure 2**).

Reproductive development is one of the most sensitive developmental stages to HS (Folsom et al., 2014; Begcy et al., 2019a). Elevated temperatures are detrimental to the formation of viable seeds due to the reduction of photo-assimilates, including starch and monomers of carbohydrates required during seed development as well as lipids used to form membranes (Zhuang et al., 2022). The photosynthetic capacity of camelina during reproductive development and seed filling stage greatly influenced the accumulation of dry matter in the plant, total seed yield and oil quality. Although physiological responses to increased temperatures are developmental stage-dependent, similar responses to high temperatures during seed development have been observed in tomato (*Solanum lycopersicum*) (Parrotta et al., 2020), rapeseed (*Brassica napus*) (Huang et al., 2019), and rice (*Oryza sativa*) (Sailaja et al., 2015).

### 4.2 Heat stress causes accelerated and altered seed development and quality

In this study we observed developmental changes in camelina seeds exposed to HS, as well as corresponding variations in lipid composition. Several aspects of plant reproduction are clearly affected by heat, such as a decreased seed set (**Figure 5**) and reduced seed size as shown by a significant decline in 100 seed weight. Previous studies have established that while *C. sativa* seed formation is completed by ~12 days after flowering, oil accumulation occurs most rapidly between 12-16 and 27-28 days after flowering and is mostly represented by the accumulation of C18:3 FA (Rodríguez-Rodríguez et al., 2013; Abdullah et al., 2016). Here, we show that seed growth under CO and HS, albeit to a lesser degree, was visible between 7 and 14 DPA. We observed browning of the CO siliques at 28 DPA, while under HS, browning was observed two weeks earlier. This clearly indicates that the period of seed development is reduced at high temperatures, thereby affecting seed weight and total yield. This accelerated but defective development is also reflected in the oil accumulation stage, which occurs between 14 DPA and 21 DPA under CO, but between 7 and 14 DPA under HS (**Figure 3**; **Table 1**). In line with our observations in *C. sativa*, decreased seed set, seed filling, and quality were also reported in oil seed crops such as *Brasicca napus*, soybean, and sunflower (Faraji et al., 2009; Izquierdo et al., 2016; Nakagawa et al., 2020; Staniak et al., 2021; Mácová et al., 2022).

### 4.3 Camelina sativa seeds exhibit altered fatty acid composition under heat stress

FAs from C4:0 to C18:0 are produced *de novo* in plastids along with mono-unsaturated FAs with Oleic acid (C18:1) being the major product of plastidial fatty acid synthesis in many oilseed species (Li-Beisson et al., 2013). Oil composition has been shown to be influenced by abiotic stressors affecting the transport of fatty acids through various organelles. Particularly, export from plastids to the endoplasmic reticulum is affected, where oleic acid (C18:1) is converted into linoleic acids (C18:2), which is further desaturated to form linolenic acids (C18:3) (Ohlrogge and Browse, 1995). A decline in the level of polyunsaturated fatty acids (i.e., C18:3) is associated with heat tolerance in cultivars of soybean and peanut (Rustgi et al., 2021), and has been shown to lead to improved heat tolerance in tobacco cells (Zhang et al., 2005; Upchurch, 2008). In this study, the quantification of FAs was performed based on lyophilized seed dry mass for greater bio preservation of samples by eliminating exposure of seeds to air and heat, which can lead to oxidation of fatty acids and degradation of PUFA (Zhuang et al., 2022). Under CO conditions, FA profile exhibited a decrease in percentage of C18:1 and C18:2 from 14 DPA onwards (**Table S1**). Correspondingly, there is an increase in the C18:3, C20:1 and other longer chain FAs in later stages of seed development (**Table 1**). Interestingly, products of FA elongase activity on MUFAs, C20:1, C22:1, and C24:1 increase drastically from 14 DPA to 21 DPA, after which changes are minor as observed previously in camelina (Rodríguez-Rodríguez et al., 2013; Vollmann and Eynck, 2015; Abdullah et al., 2016; Brock et al., 2020).

Under HS conditions, we observed plasticity in seed FA accumulation with significant qualitative and quantitative differences in composition in addition to a decrease in overall FA yield. The composition of FA over development was reversed, with the HS FA profile being more represented by C18:1 and C18:2 FAs, with a decrease in trienoic FAs suggesting that further desaturation of FAs is not preferred under HS. This observation is supported by findings that FAD3 enzymes, involved in production of PUFAs, are regulated in response to temperature, with protein degradation occurring at higher temperatures (30°C) (O’Quin et al., 2010). Similarly, the plastid C18:2 desaturase, FAD8, has been shown to undergo conformational destabilization at high temperatures of 27°C (Matsuda et al., 2005). Altogether, heat exposure can directly impact the accumulation of ω-3 FAs by impacting enzymatic activity. One factor to consider is the use of C18:3 FA in non-storage metabolic products in plants via a biosynthetic shift toward signaling. Besides being incorporated in TAGs for storage, linolenic acid is a precursor of the phytohormone jasmonic acid (JA), a biotic stress response signaling molecule which has also been shown to be involved in seed development and abiotic stress response in *Arabidopsis thaliana* (Mata-Perez et al., 2015; Balfagón et al., 2019). In fact, *fad3-2 fad7-2 fad8* Arabidopsis triple mutants exhibited male sterility, but wild type phenotype was restored by the application of JA (Wallis and Browse, 2002).

A decreased unsaturation index may be an advantageous response for several reasons. First, changes in metabolism toward stress tolerance could potentially occur via membrane modulation, whereby an increase in saturated FAs leads to a more rigid membrane. Reduction of trienoic fatty acids which are observed under HS in several plant species has been shown to confer HS tolerance to Arabidopsis double mutants *fad7fad8 (*plastidial C16:2 and C18:2 desaturases) (Murakami et al., 2000) and in *fad5* and *fad6* (plastidial C16:1 and C18:1 desaturases) (Wallis and Browse, 2002). The increase in MUFAs C16:1, C18:1, 20:1, and C22:1, especially at earlier developmental stages during seed formation, herein reported in *C. sativa* seeds under HS may be an adaptive response to HS, driven by changes in plastidial FA biosynthesis. Remodeling of membranes at this early developmental stage may aid the developing seed in surviving temperature stress, albeit at the cost of slower FA accumulation for energy storage. Interestingly, at 21 DPA, HS seeds appear to recover C18:3 accumulation, as the proportion of C18:2 FA decreases between 21 and 28 DPA, and both C18:1 and C18:2 proportion decrease between 28 DPA and maturity, while C18:3 FA accumulation is occurring between these stages. We may therefore infer that FA biosynthesis shifted to MUFA synthesis to protect cell function and embryo development by maintaining membrane stability and preventing FA oxidation, followed by a gradual return to PUFA biosynthesis. This temporary shift in lipid metabolism was also observed in the oil crop *Ricinus communis* (castor bean), which underwent a significant decrease in the polyunsaturation index of membrane lipids during HS, followed by a return to normal levels once HS was relieved (Zhuang et al., 2022). On the other hand, earlier sowing of camelina resulted in seeds with higher PUFA content due to lower temperatures during seed filling (Righini et al., 2019). Taken together, these findings support the phenotypic plasticity of camelina lipid metabolism in response to high temperature.

Here, we have described the impact of post anthesis heat on developmental FA accumulation in seeds in the biofuel crop *C. sativa* cv Suneson. The empirical evaluation of physiological performance of plants, and seed development under HS conditions compared to control aids in identification of the FA plasticity and time shift in peak FA accumulating phase in camelina due to high temperatures. Understanding the effects of temperature stress on production and quality of oil in a changing climate offer a pathway to crop improvement not only in camelina but may also be translated to other oil seed crops beyond Brassicaceae.

## Data availability statement

The original contributions presented in the study are included in the article/**Supplementary Material**. Further inquiries can be directed to the corresponding author.

## Author contributions

SN: Conceptualization, Data curation, Formal Analysis, Funding acquisition, Investigation, Methodology, Project administration, Resources, Supervision, Validation, Visualization, Writing – original draft, Writing – review & editing. LL: Formal Analysis, Investigation, Methodology, Software, Validation, Visualization, Writing – original draft, Writing – review & editing. MT: Data curation, Formal Analysis, Investigation, Methodology, Writing – review & editing. AD: Data curation, Investigation, Methodology, Software, Validation, Visualization, Writing – review & editing. AE: Data curation, Formal Analysis, Investigation, Methodology, Software, Visualization, Writing – review & editing. AS: Formal Analysis, Investigation, Methodology, Writing – review & editing. KB: Conceptualization, Formal Analysis, Funding acquisition, Investigation, Methodology, Software, Supervision, Validation, Visualization, Writing – original draft. PS: Investigation, Methodology, Validation, Writing – review & editing. CB: Conceptualization, Funding acquisition, Methodology, Writing – review & editing.

## Supplementary material

Supplementary Table 1 Percentage fatty acid (FA) composition from *Camelina sativa* seeds at various seed developmental stages calculated as % mean (*n*=5) of a FA ÷ sum of FAs ± SE. control (CO) and heat stress (HS). Asterisks denote statistically significant (SS) differences between the control (CO) and heat stress (HS) conditions at the same seed developmental stage (ns *P* > 0.05; **P* ≤ 0.05, ***P* ≤ 0.01, and ****P* ≤ 0.001) as determined by Student’s *t* tests. SE = standard error of the mean.
